# Comprehension and Understanding of Gestational Diabetes Mellitus Among Pregnant Women Attending Primary Health Care Facilities in Jeddah, Saudi Arabia

**DOI:** 10.7759/cureus.46937

**Published:** 2023-10-13

**Authors:** Reema Hakim, Ahmed Alqerafi, Waleed Malibari, Abdulaziz Allhybi, Bader Al Aslab, Alwalied Hafez, Muhannad Bin Sawad, Nawwaf Almalky

**Affiliations:** 1 Family Medicine, Bahra Primary Healthcare Center, King Abdulaziz Medical City, Jeddah, SAU; 2 Medical School, King Saud Bin Abdulaziz University for Health Sciences College of Medicine, Jeddah, SAU

**Keywords:** diabetes education, preventive healthcare, gdm- gestational diabetes mellitus, awareness, antenatal women

## Abstract

Background and objective

Gestational diabetes mellitus (GDM) is a form of diabetes that develops during pregnancy and can negatively affect both the mother and the fetus. This study aimed to assess the knowledge and awareness of GDM among pregnant women in primary care centers. By enhancing their understanding of the risks and symptoms of GDM, we can potentially mitigate adverse outcomes.

Methods

A cross-sectional study was conducted in the National Guard's primary healthcare facilities in Jeddah, Saudi Arabia. The study employed a validated 12-item questionnaire to collect data from 489 participants. The questionnaire items covered the identification of risk factors, diagnostic approach, treatment options, and complications related to GDM, considering maternal and fetal health implications.

Results

The study participants had a mean age of 30.9 years. Among these participants, 53.6% demonstrated a thorough understanding of GDM, 35.2% had moderate knowledge scores, and 11.2% had low knowledge scores. Higher levels of awareness were strongly associated with higher levels of education, gravidity, and prior knowledge of GDM.

Conclusion

The study highlights the importance of early detection and management strategies for GDM during pregnancy to minimize its negative impacts. The findings suggest the need for individually tailored antenatal education programs by healthcare professionals that address the needs of different populations. This is particularly relevant for women with lower education levels and those who are pregnant for the first time or have no prior knowledge of GDM.

## Introduction

Diabetes is a chronic disease characterized by elevated blood glucose levels [1]. Over time, it can cause substantial damage to the heart, blood vessels, eyes, kidneys, and nerves [1]. In 2019, the global prevalence of diabetes was estimated at 9.3%, with projections anticipating a rise to 10.9% by 2030 [2].

Gestational diabetes mellitus (GDM) is a subtype of diabetes, defined as glucose intolerance first identified during pregnancy [3]. As the most common pregnancy complication, GDM carries significant implications for maternal health and pregnancy outcomes. Potential maternal risks include conditions like preeclampsia and future onset of type 2 diabetes. Further, high blood sugar levels can pose serious risks to the baby's health, increasing the risk of macrosomia (large birth weight), birth complications, and congenital malformations. In Saudi Arabia, the prevalence rate of GDM is 15.5% according to a 2021 study by Al-Rifai et al. [4].

Women with a family history of diabetes mellitus, obesity, previous GDM, or rapid weight gain during pregnancy are more prone to develop GDM [5]. Screening for asymptomatic pregnant women with a low risk of GDM is recommended after 24 gestational weeks, while high-risk women should be screened before 24 weeks of gestation [6]. In the United States, the American College of Obstetricians and Gynecologists recommends the two-step test, which involves a 50g oral glucose challenge test for one hour. If the results are positive, a subsequent 100g, three-hour oral glucose tolerance test (GTT) is administered [7]. GDM is diagnosed if two or more readings are elevated. Conversely, many other countries use a one-step approach, administering a 75g GTT while fasting [8]. This test involves a 75g, two-hour oral glucose tolerance test without a preceding screening test. GDM is confirmed when one or more values exceed the threshold [7]. Non-pharmacological treatments such as exercise and dietary changes are the first line of treatment for GDM [9]. If these treatments fail to control the condition, insulin can be prescribed [10]. However, counseling and education remain pivotal in managing GDM.

Once diagnosed with GDM, pregnant women are at risk of multiple maternal and fetal adverse outcomes [11]. These complications include primary cesarean section, babies born large for gestational age, and neonatal hypoglycemia [12]. Therefore, raising GDM awareness is critical to facilitate early detection, improve management, and prevent adverse outcomes, including maternal complications such as preeclampsia and cesarean delivery, as well as neonatal complications like macrosomia. Despite GDM's high prevalence in Saudi society, few studies have assessed awareness of this condition. The primary aim of this study is to evaluate GDM awareness among antenatal women in Jeddah, Saudi Arabia.

## Materials and methods

Study settings and design

This cross-sectional study was conducted in the National Guard's Al-Waha, Iskan, and Bahra primary health care centers, in Jeddah city, Saudi Arabia, from November 2022 to March 2023. Ethical approval was obtained from the King Abdullah International Medical Research Center (KAIMRC), approval number NRJ22J/177/07, on September 27, 2023.

Study population

The antenatal clinic runs on weekdays, with an average of 12 to 28 patients attending daily. All pregnant women attending the antenatal clinic who consented to participate were included, regardless of the number of conceptions or the period of gestation. Pregnant women previously diagnosed with type 1 or type 2 diabetes were excluded. Each woman was interviewed once for data collection, and no further contact was required. The sample size was initially 377, as calculated by the Raosoft sample size calculator (Raosoft, Inc., Seattle), assuming a 95% confidence level and a 5% accepted margin of error. However, to enhance the study's robustness and representation, we included 489 pregnant women.

Data collection process

A questionnaire from a similar published study was used, with consent from the original author [13]. This questionnaire was translated into Arabic and provided in both English and Arabic, allowing participants to choose their preferred language. While the questionnaire underwent extensive review by native language professionals to ensure translation accuracy, no formal linguistic validation was performed. After obtaining informed consent, interviews were conducted using the questionnaire to guide the conversation and accurately record all required data.

The questionnaire gathered demographic information, including age, education level, parity, and previous GDM, followed by 12 questions focusing on general awareness of GDM, its risk factors, diagnosis, treatment, and consequences.

The questions on risk factors evaluated the awareness of pre-pregnancy obesity, family history of diabetes, diabetes during a previous pregnancy, and rapid weight gain during the current pregnancy. The options given were yes, no, and do not know. If the response was yes, it was considered the right answer.

A score of 1 was given for each correct answer, yielding a total score out of 12. Scores of 0-4, 5-8, and 9-12 were interpreted as poor, fair, and good knowledge of GDM, respectively.

Statistical analysis

Data were analyzed using JMP software (SAS Institute, Cary, NC). Descriptive statistics included frequency, percentage, and graphs used for categorical variables, means, and SD for continuous variables. A chi-square test for association between the categorical variables was used to examine the relations between GDM knowledge and the basic characteristics of participants. Differences in GDM knowledge by age and BMI of participants were examined using an ANOVA test. A P-value of less than or equal to 0.05 was considered significant.

## Results

Table 1 presents the demographic and clinical characteristics of the 489 antenatal women who participated in this study. The mean age of the participants was 30.9 years (SD: 6.1 years). The mean BMI was 27.9 kg/m2 (SD: 5.6 kg/m2). The majority of the participants had a university education (60.9%), followed by secondary education (28.4%) and intermediate education (10.6%). Most of the participants were multigravida (74%), and 25.6% had a previous diagnosis of GDM.

**Table 1:** Basic characteristics of participants BMI: body mass index; GDM: gestational diabetes mellitus

Variable	N = 489
Age	30.9± 6.1
BMI	27.9± 5.6
Educational level	
Intermediate	52 (10.6)
Secondary	139 (28.4)
University	298 (60.9)
First pregnancy	
Yes	127 (26.0)
No	362 (74.0)
Previous GDM	
Yes	125 (25.6)
No	364 (74.4)
Knowledge of GDM	
Yes	397 (81.2)
No	61 (12.5)
I don’t know	31 (6.3)
GDM knowledge score	8.2± 2.7

**Figure 1:**
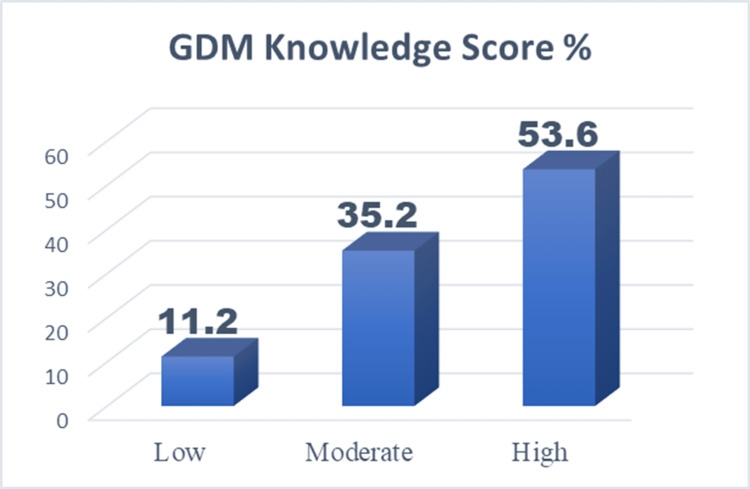
GDM knowledge score % GDM: gestational diabetes mellitus

The participants' knowledge of GDM was assessed using a validated 12-item questionnaire as summarized in Table 2. Figure 1 illustrates the distribution of knowledge scores among the 489 antenatal women. The overall results displayed a significant percentage of high scorers. Most participants (53.6%) had high knowledge of GDM, 35.2% had moderate knowledge, and 11.2% gained a low knowledge score.

**Table 2:** Awareness of antenatal women (n=489) on various aspects of gestational diabetes mellitus DM: diabetes mellitus; GDM: gestational diabetes mellitus

Aspects of Gestational Diabetes Mellitus (GDM) Awareness	number(%)
Awareness about diabetes mellitus	397 (81.1)
Awareness that diabetes can occur for the first time during pregnancy (GDM)	359 (73.4)
Awareness of the following as risk factors for GDM	
Family history of DM	391 (79.9)
Pre-pregnancy obesity	354 (72.3)
GDM in previous pregnancy	256 (52.3)
Rapid weight gain during current pregnancy	313 (64.0)
Awareness of the following aspects of diagnosis of GDM	
Time of testing as 12- 16 weeks	76 (15.5)
Time of testing as 24- 28 weeks	199 (40.0)
Test employed as blood test following an oral glucose load	300 (60.1)
Awareness of the following as treatment options for GDM	
Diet and exercise	190 (38.8)
Insulin injections	21 (4.2)
Awareness of the course and consequences of GDM	
GDM disappears after delivery	387 (79.1)
Unborn child is at risk if the mother is untreated	428 (87.5)
Women with GDM are at an increased risk for future type 2 diabetes	313 (64.0)

Table 3 presents the associations between the participants' knowledge of GDM and their basic characteristics. The participants' knowledge of GDM was significantly associated with their level of education (p < 0.001), gravidity (p < 0.001), and previous knowledge of GDM (p < 0.001). Participants with a higher level of education, multigravida women, and participants with previous knowledge of GDM had a higher knowledge score.

**Table 3:** Basic characteristics of participants by GDM knowledge score *Significant association GDM: gestational diabetes mellitus

Variable	Low	Moderate	High	P value
Educational level				
Intermediate	5 (9.6)	18 (34.6)	29 (55.8)	0.001*
Secondary	29 (20.9)	51 (36.7)	59 (42.4)	
University	21 (7.0)	103 (34.6)	174 (58.4)	
First pregnancy				
Yes	13 (10.2)	42 (33.1)	72 (56.7)	0.711
No	42 (11.6)	130 (35.9)	190 (52.5)	
Previous GDM				
Yes	6 (4.8)	32 (25.6)	87 (69.6)	0.001*
No	49 (13.5)	140 (38.5)	175 (48.1)	
Knowledge GDM				
Yes	29 (7.3)	137 (34.5)	231 (58.2)	0.001*
No	23 (37.7)	22 (36.1)	16 (26.2)	
I don’t know	3 (9.7)	13 (41.9)	15 (48.4)	

The mean age of the participants with a high knowledge score was 31.1 years (SD: 6.1 years), and the mean BMI was 28.1 kg/m2 (SD: 5.6 kg/m2) as described in Table 4.

**Table 4:** Age and BMI of participants by GDM knowledge score BMI: body mass index; GDM: gestational diabetes mellitus

Variable	Low	Moderate	High	P value
Age	30.2± 6.8	30.9± 6.3	31.1± 6.1	0.629
BMI	27.6± 5.7	27.7± 5.5	28.1± 5.6	0.666

These results suggest that most antenatal women in this study had appropriate knowledge of GDM. However, knowledge was significantly associated with level of education, gravidity, and previous knowledge of GDM.

## Discussion

This study aimed to evaluate antenatal women's knowledge of gestational diabetes mellitus (GDM) and its associated factors. The results indicate that more than half of the participants (53.6%) demonstrated a high level of GDM knowledge, while (35.2%) had moderate knowledge and (11.2%) had insufficient knowledge. This suggests that a significant proportion of the study population is well-informed about GDM, which is essential for the early detection and management of the condition.

A critical finding of this study is the association between the participants' knowledge of GDM and their level of education, gravidity, and previous knowledge of GDM. Women with higher levels of education, those who were multigravida, and those with prior knowledge of GDM demonstrated a higher knowledge score. This is consistent with previous studies that have reported a positive correlation between education level and knowledge of GDM [14,15].

The association between gravidity and GDM knowledge can be attributed to the fact that multigravida women may have had more exposure to GDM information during their previous pregnancies, either through personal experience or antenatal care. This highlights the importance of effective antenatal education for primigravida women who may lack such exposure.

The mean age and BMI of participants with high knowledge scores were 31.1 years and 28.1 kg/m2, respectively. Although this study did not investigate the influence of age and BMI on GDM knowledge, previous research has suggested that older and overweight or obese women may have an increased awareness of GDM due to their higher risk of developing the condition [16]. Further research could explore the potential impact of these factors on GDM knowledge.

The findings of this study have important implications for antenatal educational programs, particularly in terms of targeting women with lower levels of education, primigravida women, and those without previous knowledge of GDM. Tailoring educational interventions to the specific needs of these groups may help improve their knowledge and understanding of GDM, leading to better detection and management of the condition.

This study does have several limitations that warrant acknowledgment. Firstly, the use of a self-report questionnaire may have introduced a social desirability bias, as participants could potentially overestimate their understanding of gestational diabetes mellitus (GDM). Secondly, the study population was confined to antenatal women attending the National Guard's primary healthcare centers. Therefore, the findings may not be representative of the broader population. Future research could benefit from incorporating larger and more diverse samples to increase generalizability. Lastly, while the questionnaire used for data collection underwent a thorough review by native language professionals, it was not formally linguistically validated. Although interviewers were present during data collection to offer explanations and guidance to participants as required, future studies could ensure greater accuracy of results by using a linguistically validated questionnaire.

## Conclusions

In conclusion, our study reveals that a significant proportion of antenatal women possess appropriate knowledge about GDM, with their level of education, gravidity, and prior awareness of GDM serving as significant influencing factors. This highlights the importance of customized antenatal education programs that address the distinct needs of various demographic groups, particularly women with lower educational backgrounds, those experiencing their first pregnancy (primigravida women), and those without prior knowledge of GDM.

These findings strongly advocate for the development of tailored antenatal education initiatives. Such programs, designed with a nuanced understanding of different needs, could play a pivotal role in enhancing GDM knowledge among these women. This enhanced understanding could, in turn, lead to improved detection and management of GDM, thereby reducing the potential health risks associated with this condition during pregnancy. In essence, this study highlights the importance of targeted education as a vital tool in improving healthcare outcomes for antenatal women, particularly in the context of GDM management.
